# Low-dose amitriptyline versus cognitive behavioral therapy for insomnia in patients with medical comorbidity: results of a randomized controlled multicenter non-inferiority trial

**DOI:** 10.1093/sleep/zsaf176

**Published:** 2025-06-26

**Authors:** Nynke Rauwerda, Annemieke van Straten, Anouk Zondervan, Thom Timmerhuis, Marcel Smits, Pythia Nieuwkerk, Annemarie Braamse, H Myrthe Boss, Hans Knoop

**Affiliations:** Department of Medical Psychology, Amsterdam University Medical Center, University of Amsterdam, Amsterdam, The Netherlands; Department of Medical Psychology, Hospital Gelderse Vallei, Ede, The Netherlands; Department of Clinical Psychology & Amsterdam Public Health Research Institute, VU University, Amsterdam, The Netherlands; Department of Medical Psychology, Zaans Medical Center, Zaandam The Netherlands; Department of Neurology, Jeroen Bosch Ziekenhuis, ‘s-Hertogenbosch, The Netherlands; Department of Neurology, Hospital Gelderse Vallei, Ede, The Netherlands; Department of Medical Psychology, Amsterdam University Medical Center, University of Amsterdam, Amsterdam, The Netherlands; Amsterdam Public Health Institute, University of Amsterdam, Amsterdam, The Netherlands; Department of Medical Psychology, Amsterdam University Medical Center, University of Amsterdam, Amsterdam, The Netherlands; Amsterdam Public Health Institute, University of Amsterdam, Amsterdam, The Netherlands; Department of Neurology, Hospital Gelderse Vallei, Ede, The Netherlands; Department Sleep-Wake Centre, Hospital Gelderse Vallei, Ede, The Netherlands; Department of Medical Psychology, Amsterdam University Medical Center, University of Amsterdam, Amsterdam, The Netherlands; Amsterdam Public Health Institute, University of Amsterdam, Amsterdam, The Netherlands

**Keywords:** insomnia, cognitive behavioral therapy, pharmacology, group, amitriptyline, side effects, medical comorbidity, non-inferiority, discontinuation

## Abstract

**Study objectives:**

In a randomized controlled non-inferiority trial, we aimed to determine whether low-dose amitriptyline, which is often used off-label, is a safe and effective alternative to cognitive behavioral therapy for insomnia in the treatment of insomnia among patients with insomnia and medical comorbidity.

**Methods:**

A total of 187 participants with insomnia and medical comorbidity were randomly allocated to either: (1) 12 weeks of amitriptyline, 10–20 mg (*n* = 93), or (2) 12 weeks of group cognitive behavioral therapy for insomnia, seven sessions (*n* = 94). Assessments took place at baseline, 6 and 12 weeks after start of treatment. The primary non-inferiority outcome was insomnia severity (Insomnia Severity Index) at 12 weeks.

**Results:**

Based on a non-inferiority margin of four points on the Insomnia Severity Index, amitriptyline was non-inferior to cognitive behavioral therapy for insomnia at 12 weeks of treatment (mean difference of 1.1 points; 95% confidence interval = −0.5 to 2.8). Secondary analyses showed that significantly more cognitive behavioral therapy for insomnia participants reached a clinical response (≥eight-point reduction on the Insomnia Severity Index) than amitriptyline participants (58 per cent versus 41 per cent, *p* = .02). Amitriptyline participants reported more side effects (mostly anticholinergic) at 12 weeks treatment (*p* < .001) than participants who received cognitive behavioral therapy for insomnia. After discontinuation 68 per cent of the amitriptyline participants reported worsening of sleep. In 12 per cent of them this worsening was temporarily.

**Conclusions:**

With a liberal non-inferiority margin, amitriptyline is non-inferior to cognitive behavioral therapy for insomnia in reducing insomnia severity. Amitriptyline has more side effects and its effect on insomnia may diminish after tapering. Cognitive behavioral therapy for insomnia should remain first-line treatment for patients with medical comorbidity given its broader benefits.

**Statement of Significance**

Low-dose amitriptyline (AM) is an off-label treatment for insomnia and may be an effective and safe alternative to cognitive behavioral therapy for insomnia (CBT-I) in patients with insomnia and medical comorbidity. This randomized controlled non-inferiority trial demonstrated that AM is non-inferior to CBT-I in improving insomnia after 12 weeks, when considering a liberal non-inferiority margin. However, more patients showed clinically relevant improvements with CBT-I. Furthermore, AM was associated with more side effects than CBT-I and its benefits for sleep seem temporary. Therefore, CBT-I should remain the first-line treatment given its broader benefits. If patients insist on medication, AM could be considered. Then patients should be well-informed about potential side effects of AM and the likely temporary effectiveness. Our findings need replication.

## Graphical Abstract

**Graphical Abstract:**
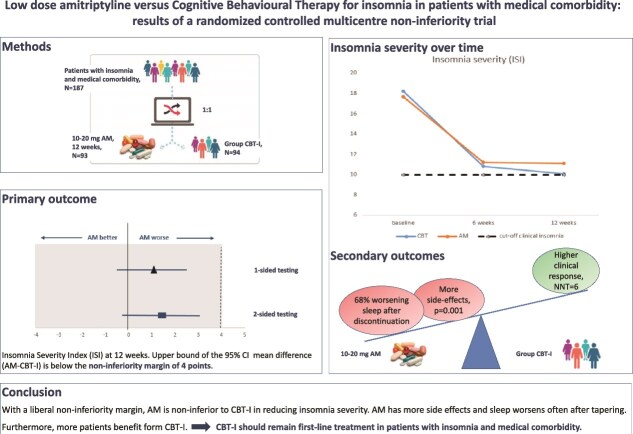

## Introduction

Insomnia disorder is prevalent in patients with long-term medical conditions [1]. Insomnia has generally been associated with an increased risk of various medical [2–4] and psychiatric disorders [5, 6], injuries [7], and decreased quality of life [8]. In patients with medical conditions sleep disorders can negatively affect the course of their comorbid medical condition [9]. Furthermore, the economic burden of insomnia with medical comorbidity is reported to be high [10]. Therefore, treating insomnia in this group is important.

Insomnia treatments can be broadly divided into pharmacological (mainly benzodiazepine receptor agonists [BZRAs]) and non-pharmacological. Clinical practice guidelines recommend non-pharmacological treatment as first choice, since the efficacy of cognitive behavioral therapy for insomnia (CBT-I) has often been shown in controlled studies [11, 12] and in comparison to BZRAs, CBT-I is more effective in the long-term [13]. Furthermore, BZRAs have disadvantages regarding the development of tolerance, dependence, adverse side effects, and rebound effects [14–16]. Preventing adverse side effects (e.g. fall risk) is particularly relevant for patients with long-term medical conditions.

CBT-I, a multicomponent treatment package, has been found to improve sleep in about 70 per cent to 80 per cent of patients [17–20], irrespective of the presence of comorbidity [21]. To enhance access to CBT-I in clinical settings, patients are often treated in a group format, which has shown to be efficacious [19]. Sustained positive effects have been found on sleep and depressive symptoms [22]. Furthermore, there is growing evidence that improved sleep following CBT-I can positively influence the clinical course of a comorbid condition [23, 24].

Despite the guidelines, BZRAs and off-label medication are still often prescribed, even at the first sleep consultation [25, 26]. One of the reasons is that clinicians often assume that patients with chronic insomnia prefer pharmacological over psychological treatment [27–31]. Clinicians often expect that patients with medical comorbidity can experience these components as strenuous or might be unable to fully engage in CBT-I due to their medical condition with associated disease burden [32]. The majority of patients prefers non-medication treatments though a small minority prefer medication [33]. Another reason is that not all insomnia patients can access CBT-I, since CBT-I is still not widely implemented [29, 34], although accessibility has improved through effective digital alternatives to (fully) face-to-face treatment [35]. Furthermore, in about 20 per cent of individuals the insomnia does not respond to CBT-I, or it remits (40 per cent) after successful CBT-I treatment [22]. There is a need for effective and safe alternatives for CBT-I that are suitable for patients with insomnia and long-term medical conditions. Given CBT-I’s established effectiveness, the alternative treatment must be at least as effective in the treatment of insomnia, supporting the use of a non-inferiority trial. One key feature of this design is that the predefined non-inferiority margin is variable depending on the method used, influencing both the interpretation of results and the study’s feasibility. A conservative margin improves sensitivity and enables non-inferiority to be shown with more certainty. However, it demands a substantial larger sample size, while a liberal margin may limit the ability to detect differences yet reduces the required sample size. To decide whether an alternative treatment can serve as a substitute for CBT-I, it is crucial to assess not only its non-inferiority in terms of its effect on insomnia severity but also compare it with CBT-I on other aspects, such as side effects.

Low-dosage (10–20 mg) amitriptyline (AM) is used as an off-label treatment for insomnia [36–38]. AM is a generic antidepressant commonly used in treatment of major depression and neuropathic pain. Patient-reported outcomes support clinical observations that low-dose AM improves sleep maintenance in the short term and is generally well tolerated [39]. Low-dose AM selectively blocks wake promoting systems via H1 receptor antagonism and thereby might improve sleep [40]. However, evidence for its hypnotic efficacy in insomnia is scarce [41]. Off-label prescription in general increases the risk of unnecessarily exposing patients to drugs without knowing how effective they are [42] and which side effects they have, which is undesirable, especially in a relatively fragile population of patients with insomnia and medical comorbidity. Therefore, a head-to-head comparison of the current off-label use of AM as sleep medication and CBT-I is needed in patients with chronic insomnia and medical comorbidity.

We aimed to determine whether AM is non-inferior to CBT-I in reducing insomnia severity among patients with insomnia and medical comorbidity. Secondary aims were to compare both interventions with respect to their effect on clinically relevant change in insomnia, subjective sleep, and daytime functioning. We also compared adherence to treatment and side effects and explored the effects of discontinuation of medication.

## Materials and Methods

### Study design

This study was a two-arm, non-blinded, multicenter non-inferiority randomized controlled trial (RCT) in which the efficacy of AM was compared with the gold standard CBT-I. The study was carried out in the outpatient clinics of the departments of neurology/sleep–wake centers and medical psychology of four general hospitals in the Netherlands. After providing written informed consent, eligible patients were randomized in random blocks (2 or 4) in a ratio of 1:1 to either (1) group CBT-I or (2) low-dose AM. Randomization was stratified by benzodiazepine use at baseline (i.e. no versus benzodiazepine use at least once every 2 weeks) and by referral type (self-referral yes/no). Randomization was computer generated using an online structural database (CastorEDC) and was executed centrally by the coordinating researcher. Allocation was therefore blinded. Participants or therapists were not blinded for treatment allocation; statistical analysis was conducted by an independent statistician who was blinded for group allocation. Participants completed self-report measures at baseline (before randomization) and at 6 and 12 weeks after start of treatment. The latter was the primary endpoint of the study. Participants who received AM were also assessed at 14 weeks to determine the effect of discontinuation after week 12. The trial was registered in the Dutch Trial Register (NTR NL7971, August 18, 2019). Ethics approval was obtained from the Medical Ethics Committee of Amsterdam University Medical Center, University of Amsterdam (2019_101, July 17, 2019), and the ethics committees of the participating hospitals. Reporting follows CONSORT guidelines [43, 44]. For a detailed description of the study, we refer to the published protocol [45]. The protocol is available upon request by emailing the Principal Investigator.

### Participants

Patients who visited the sleep clinic (upon referral by the general practitioner or medical specialist) of the participating hospitals and met the inclusion criteria were informed by their neurologist about the study and invited to participate. Inclusion criteria were: (1) being diagnosed with a long-term medical condition as confirmed by the physician assessing the patient, (2) age between 18 and 85 years, and (3) suffering from insomnia disorder meeting DSM-5 criteria, and scoring ≥10 on the Insomnia Severity Index (ISI) [46]. We defined a long-term medical condition as a chronic medical condition and/or persistent physical complaints (>3 months) that (1) requires medical attention (e.g. visiting an outpatient clinic and receiving some form of treatment) and (2) causes dysfunction, discomfort, or social problems. For exclusion criteria, see Table 1. In case, patients were informed outside the care provision (e.g. by social media) and wanted to participate (self-referral), they had to be referred by their general practitioner or medical specialist to one of the participating hospitals to check eligibility.

**Table 1:** Inclusion and exclusion criteria

Inclusion criteria	Exclusion criteria
18–85 years Insomnia disorder conform DSM-5 Score of ≥10 on the Insomnia Severity Index Long term medical condition	Habitual night shifts Untreated sleep related breathing disorder Wish to continue over-the-counter sleep aids Off-label amitriptyline for insomnia in past year Epilepsy, dementia, history of delirium Being unable to follow study instructions and fill out the study questionnaires in Dutch Terminal illness (life expectancy <1 year) Severe psychiatric disorder Substance abuse/addiction (benzodiazepine allowed)
	Potential drug–drug interactions for amitriptyline: Use of psychopharmaceuticals (other than benzodiazepine) or antimycotics (e.g. antidepressants, anticonvulsants)
	Contra-indications for amitriptyline: Allergy for amitriptyline Pregnancy, lactation, or pregnancy wish Ocular hypertension/glaucoma Cardiac disorders Severe renal insufficiency or liver dysfunction

### Interventions

During the treatment period, all participants had access to written or online sleep hygiene advice. Participants were allowed to receive care for their medical condition, but were instructed to refrain from using over-the-counter treatments as much as possible. The general practitioner and the pharmacy of the participant were informed about study participation so that new prescriptions were checked for potential drug–drug interactions.

#### Amitriptyline

The treatment regimen for AM mimicked current off-label practice as much as possible with a 12-week treatment period, starting with 10 mg AM daily before going to bed and after 3 weeks the possibility to double the dose to 20 mg daily and return to 10 mg daily based on perceived effect and side effects (“self-titration”) [39]. Discontinuation was at 12 weeks treatment. If preferred, the participants could use a tapering schedule of maximally 2 weeks. All participants in the AM group had a consultation with a neurologist at 6 and 12 weeks to evaluate the effect, dosage, and side effects of treatment and discuss the discontinuation of medication at 12 weeks.

#### Cognitive behavioral therapy for insomnia

Reporting is according to the TIDieR checklist [47]. CBT-I consisted of 6 weekly group sessions (75–120 min depending on the group size varying between two and eight participants) and a booster session at 12 weeks. Nine psychologists, all specialized and trained in insomnia, CBT-I, and medical psychology, led the sessions. Four of them were junior psychologists, supervised by an experienced clinical psychologist, and five were licensed clinical psychologists. Participants were scheduled into groups and therefore could not start treatment immediately following randomization. The CBT-I manual of Verbeek et al. [48] was used and treatment elements were: psycho-education, sleep hygiene, relaxation, stimulus control and sleep restriction, cognitive interventions and relapse prevention. Therapists followed the structured written manual to ensure treatment components were applied consistently across therapy groups. Participants received the corresponding workbook [49], which outlined the exact steps of the manual, ensuring that all participants received the elements of the CBT-I. From session 2 onwards, sleep restriction was tailored, adapted to the patient’s progress and individual needs. Treatment was offered face to face at the site of participating hospitals. During the measures of the COVID-19 pandemic, the group treatment was offered via videoconferencing in group or individually via a secured connection [50]. No integrity checks were carried out.

### Outcome measures

Data were collected and stored digitally using a structural database (CastorEDC). This system allows no missing items and sends reminders automatically. In case, participants preferred a paper version of the questionnaires, this was sent by regular mail and the completed forms were stored anonymously and safely.

#### Insomnia severity

Insomnia severity, the primary outcome, was measured with the ISI [46]. The ISI is a seven-item questionnaire scored on a five-point Likert scale reflecting the severity of both nighttime and daytime aspects of insomnia disorder as perceived by the participant in the last 2 weeks. Total scores range from 0 (no insomnia) to 28 (severe insomnia). A score of ≥10 was used to define clinical insomnia [51]. A treatment response was defined as a reduction from baseline to 12 weeks after the start of the treatment of ≥8 points [46].

#### Subjective sleep

An online version of the Consensus sleep diary [52] was used for 1 week to assess subjective sleep characteristics. Sleep diary outcomes included: sleep efficiency (SE), i.e. percentage of time slept from the total amount of time spent in bed; sleep onset latency (SOL), i.e. time it takes to first fall asleep; wake after sleep onset (WASO), i.e. time awake after first sleep onset; number of awakenings (NOA); and total sleep time (TST). For each variable a mean score was calculated. Data were assumed missing when <4 days were registered.

#### Daytime functioning

Daytime functioning was assessed with the following self-report measures: (1) The eight-item *subscale Fatigue severity of the Checklist Individual Strength (CIS-fat*) to assess fatigue in the previous 2 weeks. A total score (range of 8–56) was computed. A score of 35 or higher indicates severe fatigue [53]. (2) *Hospital Anxiety and Depression Scale (HADS)* [54, 55] to assess anxiety and depression. The HADS contains 14 items (7 on depression and 7 on anxiety). A total score (range of 0–21) for each subscale was computed. Higher scores indicate more severe anxiety and depression and a score of ≥8 on the subscales indicates moderate symptoms of either anxiety or depression. (3) The Subscale *interference of bodily pain of the Short-Form 36-item Health Survey (SF-36-int.pain)* was used to assess the interference of pain on daytime functioning. The item interference of pain ranges from 0 to 50. Higher scores indicate less interference [56]. (4) The Subs*cale physical functioning of the SF-36 (SF-36-PF)* [56] was used to assess physical functioning. A total score from 10 items was calculated ranging from 0 to 100. A high score defines more favorable physical functioning. (5) The *Work and Social Adjustment Scale* [57] was used to asses impairment of functioning (work, home management, social leisure activities, private leisure activities, close relationships). A total score was computed (range of 0–40), with a higher score representing more impairment.

#### Treatment adherence

Adherence to CBT-I was assessed by the number of attended sessions and a posttreatment five-item questionnaire at 6 weeks. This questionnaire assesses, on a five-point scale, how well participants followed each of their five treatment instructions over the past weeks. These instructions include sleep hygiene advice, relaxation, use of a sleep diary, behavioral interventions (sleep restriction and stimulus control), and cognitive interventions (worry program and thought diary). Item scores were added up (range of 5–25), higher scores representing better adherence.

Treatment adherence to AM was measured by pill count and a five-item questionnaire (the Medication Adherence Rating Scale [MARS-5]) [58] at 12 weeks. Each of the five items is rated on a five-point Likert scale. Item scores were added up (range of 5–25), higher scores representing better adherence. Adherence by pill count was calculated by the number of tablets provided minus the number of tablets returned to the investigator or reported as lost divided by the number of days on the study (correcting for dose) multiplied by 100.

#### Side effects

Side effects were assessed with the following self-report measures: (1) The Antidepressant Side-Effect Checklist (ASEC) [59] to assess common side effects of antidepressants. We split symptom 2 “Drowsiness” into “difficulty waking up, drowsiness when waking up,” and “drowsiness during the day.” Symptom 3 “Insomnia” (difficulty sleeping) was split into “vivid dreams” and “disturbed sleep” as these might be side effects of AM. Room for comments and reporting of additional side effects was provided. A total score of the 24 items was computed, with higher scores indicating more side effects. (2) The Epworth Sleepiness Scale (ESS) [60] to asses daytime sleepiness (as side effect). The ESS is an eight-item questionnaire (scores 0–24) that requires participants to indicate how likely they would be to fall asleep or doze in various situations. ESS scores >10 are considered to indicate excessive levels of daytime sleepiness.

Both self-report measures were assessed at baseline and 6 and 12 weeks of treatment. Participants were asked to report any adverse event during consultation or in between by contacting the researcher.

#### Discontinuation

At 12 weeks all participants were instructed to discontinue or taper off AM. Withdrawal symptoms were measured 2 weeks later with the 43-item Discontinuation-Emergent Signs and Symptoms (DESS) checklist [61], adjusted to a self-administration format. Participants were asked whether they had newly experienced one of the listed signs or symptoms during the first week after they discontinued the treatment. The number of newly occurring DESS events was used to measure the effect of discontinuation. Furthermore, participants were asked how they tapered off the medication and whether they experienced a worsening of sleep.

#### Additional measures

Current and previous use of sleep medication was assessed by the neurologist-somnologist and registered in the electronic patient file/record. Patient characteristics (i.e. sex, age, BMI, marital status, highest attained educational level, current work status, smoking status, alcohol consumption, use of over the counter drugs [OTCs], duration of insomnia) were assessed in a self-constructed questionnaire.

### Statistical analysis

Statistical analyses were performed using SPSS 28.0 (SPSS Inc., Chicago, IL, USA). Demographic and clinical characteristics, as well as primary and secondary outcomes at baseline, are presented in means (standard deviations) and numbers (percentages) depending on their distribution.

The null hypothesis stated that the effect of AM on the primary outcome is worse than CBT-I by more than the pre-specified non-inferiority margin. The alternative hypothesis was that AM is not worse than CBT-I by more than the pre-specified non-inferiority margin of four points on the ISI. For defining the non-inferiority margin, we used the anchor based method, i.e. 50 per cent of the minimum clinically significant difference reported following CBT-I. Based on research, the minimum clinically significant difference is 8 points [47].

To test for the difference between the two treatment groups, we compared the primary outcome of ISI score at week 12 between treatment groups (AM or CBT-I) using analysis of covariance (ANCOVA) adjusted for the baseline ISI score. We checked for violations of necessary assumptions of ANCOVA. We calculated a one-sided 95% confidence interval (CI) around the mean difference in the primary outcome between the treatment groups to determine if the upper limit of the confidence interval was below the non-inferiority margin of four points. AM was declared non-inferior to CBT-I if the upper bound of the 95% CI did not exceed the non-inferiority margin of four points. This primary analysis was conducted according to intention-to-treat with imputed data to handle missing values. We used multiple imputations using chained equations to impute missing data. We included all baseline demographic and clinical characteristics in the imputation model. We imputed a total of five datasets and these were pooled according to Rubin’s rule [62]. We repeated the primary analysis as a complete case analysis and as per protocol analysis. In the latter analysis, only participants who actually received their assigned treatment (in the CBT-I group attendance of at least five out of seven sessions and in the AM group at least 70 out of 84 days medication) were included.

For all further analyses, with the exception of mixed model analyses, a complete case approach was used, using ANCOVA and including only patients with no missing data on the relevant variables. For secondary analyses involving mixed models, missing data were estimated using available data from other time points, as these models can handle missing data more flexibly.

If AM would show to be non-inferior, we would proceed to superiority hypothesis testing. For superiority testing, we considered the two sided 95% CI of the mean difference between both groups on the primary outcome as established in the ANCOVA and verified if it included zero or not. We decided to conduct an additional analysis in order to compare the percentage responders post treatment, i.e. a ≥8 point reduction in ISI score between both treatment groups using logistic regression analysis. This outcome was expressed as the number needed to treat (NNT) as well. NNT was computed as the inverse of the absolute risk difference between AM and CBT-I.

During the COVID-19 pandemic, participants received CBT-I via videoconferencing instead of face-to-face. In a post hoc analysis, we repeated the comparison of insomnia severity between both groups, restricting the analyses to participants who received face-to-face CBT-I.

Participants were permitted to use BZRAs during treatment. In a post hoc analysis, we repeated the non-inferiority analysis, restricting it to participants who did not use BZRAs during treatment.

Additionally, we compared the mean ISI score and the subjective sleep outcomes over time between the two treatment groups using a linear mixed model with treatment group (AM or CBT-I), time (baseline, 6 and 12 weeks) and an interaction-effect (group × time) included as fixed effects and a random intercept to account for repeated measurements within participants.

We compared the secondary outcome measures on daytime functioning assessed at week 12, between both treatment groups, adjusted for the baseline measurements using ANCOVA. We compared side effects (as assessed with the sum score of the ASEC and of the ESS) at week 6 and week 12 between both groups using ANCOVA adjusted for baseline score. Rates of adherence and withdrawal (for AM) are described. Bonferroni corrections were applied to the *p*-values to account for multiple comparisons.

### Sample size

The SD of the ISI in our study was assumed 4.4 [63]. Sample size calculation following the recommendations outlined in Tamayo-Sarver et al [64] using a one-tailed test and a 5 per cent significance level, showed that 84 participants in each group would provide adequate power (90 per cent) to reject the null hypothesis that the ISI change brought on by low-dose AM is worse to that brought on by CBT-I, with a non-inferiority margin of four points. The dropout rate was estimated to be 13 per cent (41). Hence, 190 participants in total had to be included.

## Results

**Figure 1:**
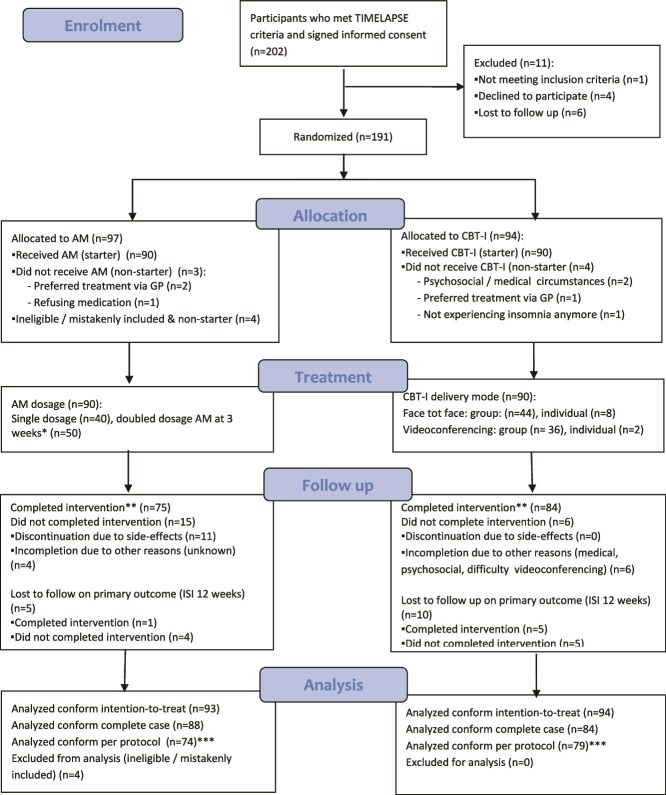
Flowchart of enrolment and randomization.

Participants were included between September 3, 2019 and March 15, 2024. We stopped recruitment when we reached our originally intended sample size. At the time of submission, the 1-year follow-up after treatment was still ongoing. It is unknown how many patients were informed about the study. Figure 1 presents the participant flow through the study. Of the referred patients, 202 were eligible and signed informed consent. Of them, 191 were randomized (see Figure 1). Of the randomized participants, 23 were self-referrals (12 per cent). Four (of 94) participants randomized to CBT-I did not start the intervention. Four (of 97) participants randomized to AM were mistakenly included (not meeting TIMELAPSE criteria). These latter four participants were excluded after randomization but before the start of the intervention. Apart from these four excluded participants, three (of 93) participants did not start with the medication.

Table 2 presents the demographic, lifestyle, and clinical variables of the participants pretreatment. The sample (*n* = 187) was mostly female (59 per cent), predominantly mid-to highly educated (86 per cent) and 60 per cent were currently employed. The mean BMI was 26.9. Of the participants, 100 (53 per cent) had 1 medical comorbidity and 87 (47 per cent) had 2 or more medical comorbidities. The most prevalent medical comorbidities were endocrine, nutritional, or metabolic diseases (*n* = 50), symptoms, signs, or clinical findings, not elsewhere classified (*n* = 41), diseases of the nervous system (*n* = 39), comorbid sleep wake disorders (*n* = 36), diseases of the respiratory system (*n* = 27), and diseases of the musculoskeletal system and connective tissue (*n* = 25) (see also Supplementary Appendix A). Of the participants, 56 (30 per cent) used BZRAs as sleep medication.

**Table 2:** Descriptive statistics of baseline sample characteristics

	Total *n* = 187	CBT-I *n* = 94	AM *n* = 93
Demographic variables			
Age, *M* (*SD*)	52.7 (12.3)	53.3 (12.7)	52.1 (11.9)
Female, *N* (%)	111 (59.4)	55 (57.9)	56 (60.2)
BMI, *M* (*SD*)	26.9 (5.0)	27.1 (5.3)	26.7 (4.7)
Education			
High, *N* (%)	88 (47.1)	45 (47.9)	43 (45.3)
Middle, *N* (%)	73 (39.0)	36 (38.3)	37 (41.1)
Low, *N* (%)	25 (13.3)	13 (13.8)	12 (13.7)
Marital status: partner, *N* (%)	144 (77.0)	77 (81.9)	67 (72.0)
Occupational status: employment, *N* (%)	112 (59.9)	50 (44.2)	62 (66.7)
Lifestyle variables			
Caffeine use, *N* (%)	138 (73.8)	75 (79.8)	63 (67.7)
Alcohol use, *N* (%)	103 (55.1)	51 (54.3)	52 (55.9)
Smoking, *N* (%)	15 (8.0)	7 (7.4)	8 (8.6)
Insomnia related variables			
Insomnia severity (ISI), *M* (*SD*)	18.0 (3.5)	18.2 (3.5)	17.7 (3.5)
Duration insomnia (years), *M* (*SD*)	11.2 (11.5)	12.7 (11.8)	9.7 (11.0)
Use of (BZRAs) for sleep, *N* (%)	57 (30.5)	29 (30.5)	27 (29.0)
Clinical variables			
Number of medical comorbidities, *M* (*SD*)	1.6 (0.8)	1.7 (0.8)	1.6 (0.9)

*M* and *SD* are presented for continuous variables, and *N* (%) are presented for categorical variables.

### Treatment implementation

After randomization, AM participants were able to start their treatment sooner (*M* = 12.8 days; *SD* = 18.1) than CBT-I participants (*M* = 23.7 days; *SD* = 19.6; *p* < .01). Of the 90 AM participants, 50 (56 per cent) participants doubled their dosage during treatment. Due to COVID-19 regulations, 38 participants (42 per cent) could not receive face-to-face CBT-I and therefore received CBT-I via videoconferencing, with 36 participating in group sessions and two in individual sessions. There were no statistically significant differences in the use of BZRAs between the two groups during treatment (AM: 14 per cent versus CBT-I: 15 per cent, *p* = .79), or use of OTCs such as valerian (AM: 4 per cent versus CBT-I: 6 per cent, *p* = .52).

#### CBT-I treatment adherence

The majority of participants attended all seven CBT-I sessions (*n* = 60, 64 per cent). Of the 90 participants, 84 (93 per cent) completed at least five of seven treatment sessions. The mean number of sessions was 6.0 (*SD* = 1.9). When a session was missed, it was most often the seventh session (40 per cent of the missed sessions), a booster session with repetition of all the elements of CBT-I. The mean sum score of the Questionnaire homework CBT-I (range of 0–25) was 18.3 (*SD* = 3.9). The mean adherence to the element cognitive therapy was 2.4 (*SD* = 1.1; range 1 = never to 5 = always). The mean adherence to the other four elements was: sleep hygiene 4.0 (*SD* = 1.2), sleep wake registration 4.2 (*SD* = 1.3), behavioral techniques 4.1 (*SD* = 1.2), and relaxation 3.7 (*SD* = 0.9).

#### AM treatment adherence

The mean days of taking AM was 79.5 (*SD* = 23.6). Of the 90 participants, 75 (83 per cent) completed treatment (at least 70 out 84 days). The mean (*SD*) percentage medication adherence by pill count was 95.1 (*SD* = 11.3). The mean sum score of the MARS-5 (range of 0–25) was 23.3 (*SD* = 2.6), with a higher score indicating a better adherence.

### Insomnia severity

#### Non-inferiority analyses

**Figure 2:**
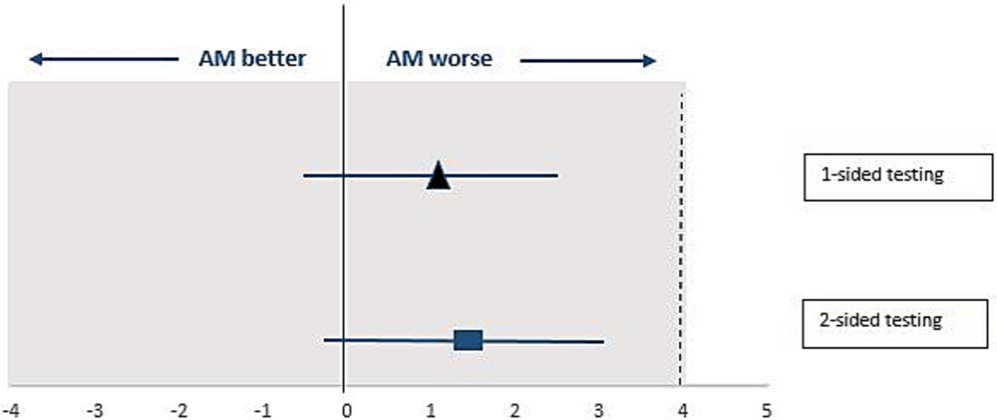
Difference between AM and CBT-I group (AM minus CBT-I) in mean Insomnia Severity Index score (95% CI) at 12 weeks according to non-inferiority and superiority testing.

The mean difference between the AM and CBT-I groups in insomnia severity at week 12 was 1.1 points (95% CI = −0.5 to 2.8) (see Figure 2). This difference did not exceed the non-inferiority margin of four points. The results from the analysis without imputed data (complete case analysis) were similar, with the mean difference between the groups remaining within the non-inferiority margin of four points. Also in per-protocol analysis (AM, *n* = 74, CBT-I, *n* = 79), the difference in insomnia severity between both groups (mean difference of 0.9 points (95% CI = −0.6 to 2.4) did not exceed the non-inferiority margin. In a post hoc analysis restricting the sample to participants not using BZRAs during treatment, no different conclusions were reached: the mean difference in insomnia severity between the AM and CBT-I groups at week 12 was 1.2 points (95% CI = −0.3 to 2.7).

#### Secondary analyses

AM was not superior to CBT-I (mean difference 95% CI = 1.4 (−0.2 to 3.1), *p* = .09) (see Figure 2). At 12 weeks of treatment, significantly more CBT-I participants reached a clinical response (≥8 points reduction on the ISI) than AM participants (CBT-I: *n* = 49 (58 per cent) versus AM: *n* = 36 (41 per cent), OR = 2.02, *p* = .02). The NNT in favor of CBT-I in comparison to AM was 6.

#### AM versus CBT-I face to face

When considering only patients in whom CBT-I was delivered in a face-to-face format (*n* = 52), the mean difference between AM and CBT-I still did not exceed the non-inferiority margin, with a mean difference of 2.3 (95% CI = 0.7 to 3.9). CBT-I was, however, now superior to AM (mean difference 95% CI = 2.3 (0.4–4.2), *p* = .02). At 12 weeks treatment, more CBT-I participants reached a clinical response (≥8 points reduction on the ISI) than AM participants (CBT-I 69 per cent versus AM 41 per cent, OR = 3.25, *p* = .001). The NNT in favor of CBT-I in comparison to AM was 4.

#### Change in insomnia severity over time

**Figure 3:**
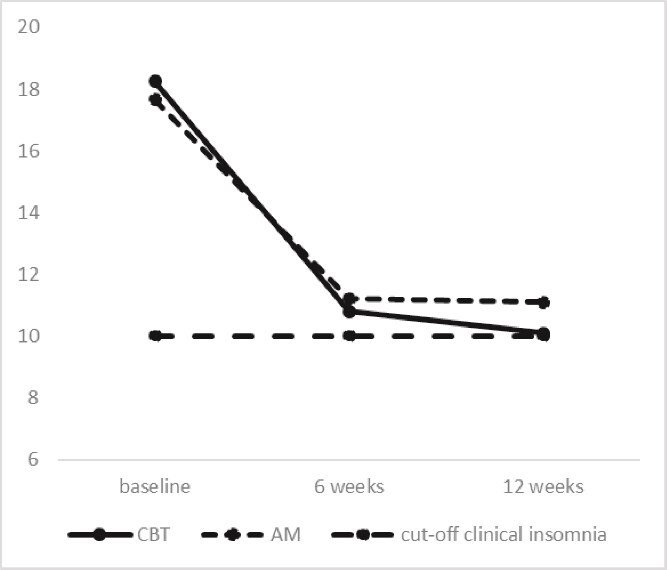
Insomnia severity over time as measured with the ISI at baseline, 6 and 12 weeks treatment.

CBT-I and AM did not differ in pattern of change in insomnia severity score over time (group by time interaction: *p* = .18) (see Figure 3).

### Subjective sleep

More AM (*n* = 85) than CBT-I participants (*n* = 66) completed the sleep diary at 12 weeks treatment (91 per cent versus 70 per cent *p* < .001) (see Table 3). In participants treated with AM TST increased more over time than in participants treated with CBT-I (group × time effect, *p* = .03) (see Table 3). No differences between participants treated with CBT-I and AM over time were found regarding SOL, WASO, NOA, and SE. In both groups, SOL and WASO decreased and SE increased over time (*p* = .01, *p* < .001, and *p* < .001, respectively), mainly between baseline and 6 weeks treatment (see Figure 3).

**Table 3:** Sleep diary outcomes over time in patients receiving CBT-I or AM

Outcome measure	Treatment	*N*	Pretreatment Mean (*SE*)	*N*	6 weeks Mean (*SE*)	*N*	12 weeks Mean (*SE*)	*P* Time-effect	*P* Group × time effect
Sleep diary									
Total sleep time	CBT-I	93	5.9 (0.2)	68	5.8 (0.1)	66	6.2 (0.1)	.02	.03
	AM	92	6.0 (0.2)	81	6.8 (0.2)	85	6.8 (0.2)		
Sleep onset latency (min)	CBT-I	93	49.3 (5.0)	68	28.5 (4.4)	66	33.8 (5.0)	.01	.70
	AM	92	54.5 (5.0)	81	41.6 (4.0)	85	43.1 (4.3)		
Wake after sleep onset (min)	CBT-I	93	85.4 (6.9)	68	60.7 (7.2)	66	55.8 (7.2)	<.001	.88
	AM	92	89.1 (6.9)	81	61.4 (6.6)	85	63.4 (6.4)		
Number of awakenings	CBT-I	93	3.5 (0.2)	68	3.2 (0.2)	66	3.0 (0.3)	.08	.52
	AM	92	3.8 (0.2)	81	3.2 (0.2)	85	3.7 (0.3)		
Sleep efficiency (%)	CBT-I	93	71.2 (1.8)	68	78.7 (2.0)	66	80.0 (2.0)	<.001	.86
	AM	92	70.2 (1.8)	81	79.1 (1.9)	85	79.3 (1.8)		

^*^Data were assumed missing when <4 days were adequately registered. Of those not completing the sleep diary in the AM group (*n* = 7): lost to follow up (*n* = 2), non-responders (change ISI from baseline <8 points: *n* = 4), and responder (change ≥8 points, *n* = 1). Of the non-completers in the CBT-I group (*n* = 27) lost to follow up (*n* = 9), non-responders (*n* = 10), and responders (*n* = 8).

### Daytime functioning

At baseline, participants reported high levels of fatigue (CIS-fatigue ≥35), moderate levels of anxiety (HADS-anx ≥8), and low levels of depressive symptoms (HADS-dep < 8). No differences were found between both groups on any of the daytime functioning outcomes, i.e. fatigue, physical functioning, interference of pain on daytime functioning, anxiety, depressive symptoms, and impairment on social functioning, assessed at week 12 and adjusted for baseline measurements (see Supplement 1).

### Side effects

Table 4 presents reported side effects as measured with the ASEC and ESS at baseline and 6 and 12 weeks treatment. There were no significant differences between both groups in reported side effects as measured with the ASEC sum-score at week 6 when adjusted for baseline scores (*p* = .2). At 12 weeks, AM participants reported more side effects as measured with the ASEC sum severity-score than CBT-I participants (10.6 versus 7.4) when adjusted for baseline scores (*p* = .001). AM participants reported the following complaints more frequently during treatment versus pretreatment: dry mouth, difficulty waking up, vivid dreaming, increased appetite, difficulty urinating, and sweating. There were also symptoms that were reported less frequently than pretreatment, such as headache and restless sleep. CBT-I participants reported almost all symptoms less frequently during treatment versus pretreatment. See Supplements 2 and 3 for a detailed overview per symptom of the ASEC. No excessive levels of daytime sleepiness were reported (ESS > 10), in neither the CBT-I nor the AM group. There were no significant differences between groups in reported daytime sleepiness as measured with the ESS sum-score at weeks 6 and 12 when adjusted for baseline score (*p* = .1 and *p* = .2, respectively).

**Table 4:** Side effects

Outcome measure	Treatment	*N*	Pretreatment Mean (*SE*)	*N*	6 weeks treatment Estimated marg. Mean (SE)^*^	Mean between group difference (95% CI)	*P*	*N*	12 weeks treatment Estimated marg. Mean (SE)^*^	Mean between group difference (95% CI)	*P*
Symptoms of common antidepressant side effects											
ASEC	CBT-I	93	14.6 (0.8)	74	9.7 (0.7)	1.6 (−0.4 to 3.5)	0.2	73	7.4 (0.7)	3.2 (1.2–5.2)	.001
	AM	92	14.0 (0.8)	83	11.2 (0.7)			81	10.6 (0.7)		
Daytime sleepiness											
ESS	CBT-I	94	4.3 (0.3)	75	3.9 (0.2)	−0.6 (−1.2 to 0.1)	0.09	74	3.1 (0.2)	0.5 (−0.2 to 1.1)	.15
	AM	93	3.8 (0.3)	86	3.4 (0.2)			85	3.6 (0.2)		

^*^Controlled for sum of symptom severities at baseline.

ESS scores >10 are considered to indicate excessive levels of daytime sleepiness.

### Discontinuation of AM

Of the 90 participants starting AM, 11 (12 per cent) discontinued AM prematurely due to side effects (depressive complaints, hallucinations, dizziness, palpitations, worsening of insomnia, illness, and nausea). Of the 79 participants using AM for approximately 12 weeks, 4 participants were not willing to taper off and continued using AM after 12 weeks. The DESS checklist was filled out by 73 (97 per cent) of the 75 participants who discontinued AM treatment at 12 weeks treatment (see Supplementary Appendix B for the results). Most AM participants (63 per cent) experienced complaints after discontinuation (participants using 10 mg: *n* = 17, 61 per cent, 20 mg: *n* = 29, 64 per cent). The five most reported complaints after discontinuation were fatigue (*n* = 14), nervousness (*n* = 12), headache (*n* = 10), frequently dreaming/nightmares (*n* = 9), and restless legs (*n* = 9). Most participants (*n* = 50, 68 per cent) reported worsening of sleep (10 mg, *n* = 20, 71 per cent, 20 mg, *n* = 30, 67 per cent); of them, a small proportion (*n* = 6, 12 per cent) reported this worsening was temporarily.

## Discussion

We investigated whether low-dose AM is an effective and safe alternative for CBT-I for patients with insomnia and co-morbid long-term medical conditions. We found that 12 weeks treatment with AM was non-inferior to group CBT-I in reducing insomnia severity. Insomnia severity decreased significantly in both treatment groups, particularly between the start and 6 weeks of treatment and the difference between both conditions fell within the non-inferiority margin. However, significantly more patients in the CBT-I condition reported a clinically relevant reduction of insomnia than patients receiving AM. In AM more improvement was found on subjective TST. No differences were found between treatments in their effects on other subjective sleep characteristics and daytime functioning. Patients treated with AM reported more side effects than patients treated with CBT-I. After discontinuation most participants treated with AM reported a return of sleep problems.

This is the first head-to-head comparison of AM and CBT-I in an adequately powered RCT. Our primary study hypothesis was supported since the mean difference did not exceed the non-inferiority margin, indicating that AM is non-inferior to group CBT-I in the treatment of insomnia in patients with medical conditions. This finding is in line with a previous uncontrolled study, which found that low-dose AM improves subjective sleep on the short term (6 weeks treatment) [40]. In that study, only participants with sleep maintenance problems were included. In the present study, we included participants with insomnia and long-term medical conditions irrespective of whether they experienced sleep onset problems, sleep maintenance problems or early morning awakenings.

Our results are based on our predefined non-inferiority margin of four points on the ISI. We determined the margin using an anchor-based method, which corresponds to 50 per cent of the minimally clinically relevant difference on the ISI reported following CBT-I. Non-inferiority margins in insomnia trials range from 1.5 to 4 points on the ISI, depending on the methodological approach used [65–67]. For instance, a distribution-based method would yield a more conservative margin. The four-point margin employed in this study can be considered liberal. This liberal margin made it feasible to conduct the study, given the magnitude of the required sample size, but is less sensitive to detect differences. With more stringent margins, like the distribution-based method, AM would not be classified as non-inferior to CBT-I.

Although mean ISI scores in the AM group remained within our predefined non-inferiority margin, significantly fewer participants benefitted clinically from AM compared to group CBT-I. The disparity was even greater when considering only face-to-face CBT-I participants. The high treatment response for face-to-face CBT-I aligns with previous research [20], whereas the overall CBT-I response rate in our study was lower. Due to COVID-19 restrictions, we temporarily could not offer face-to-face group CBT-I; instead, participants received treatment via videoconferencing (~40 per cent of the sample), predominantly in group format. While individual CBT-I via videoconferencing has shown efficacy [51], group videoconferencing format effectiveness remains uninvestigated. When the subgroup of patients who received CBT-I face to face was compared to the total group of patients who received AM, there was a significant larger decrease in insomnia severity which was not the case when all patients were analyzed.

Our study showed that the most significant improvement for both treatments occurred within the first 6 weeks. Previous research also found that optimal outcomes for CBT-I were achieved relatively fast [68], with 4–8 weekly sessions recommended for best results [12]. Similarly, we observed these rapid improvements with AM.

CBT-I participants experienced a delay in starting treatment due to scheduling in groups, with an average waiting period of about 11 days. However, being able to start directly with AM did not result in a superior outcomes of AM compared to the group format of CBT-I. Alternative CBT-I formats, such as individual therapy or digital options [51], can lead to a shorter waiting period for CBT-I.

In our study, the AM participants had access to written sleep hygiene advice, which is a basic element of CBT-I. Although the effectiveness of sleep hygiene advice is low [69] and we do not know how many individuals followed the recommendations, we cannot rule out the possibility that it contributed to the improvements observed in the AM group.

Patients treated with AM reported significantly longer (~30 min) subjective TST than patients treated with CBT-I as reported on a sleep diary. The increased subjective TST with AM has also been found in a previous study in a non-clinical sample, although not differing from placebo [70]. Further placebo-controlled studies are necessary to investigate the effect of a low-dosage AM on subjective TST. Our analyses of the sleep diaries from participants treated with CBT-I showed only a small increase in TST by the end of treatment, consistent with previous research [71]. The subjective perception of increased TST after AM may account for the reduction in insomnia severity among AM participants. CBT-I is known to improve insomnia severity through mechanisms beyond extending subjective sleep time, such as addressing sleep-related safety behaviors and dysfunctional sleep related beliefs [72, 73]. Future studies should investigate the distinct mediating mechanisms influencing insomnia severity in AM in comparison to CBT-I.

Participants treated with AM and CBT-I showed similar improvements on the subjective sleep characteristics, SOL, WASO, and SE. In both groups, participants reported an improvement of SOL of ~15 min. Improvements on WASO for both groups were ~30 min and SE increased with almost 20 per cent. These improvements are in line with improvements found in previous studies in patients with insomnia and medical comorbidity [74] treated with CBT-I. Furthermore, improvement on subjective SOL was relatively similar to improvements found with short treatment BZRA [75].

At baseline, participants reported severe fatigue, which is not uncommon in individuals with insomnia and long-term medical conditions [76]. Fatigue is usually an important factor why patients with insomnia seek help [77, 78]. No differences were found post treatment between groups on any outcomes related to daytime functioning, including fatigue. Given that fatigue is a significant issue for individuals with insomnia and long-term medical conditions, future studies should prioritize fatigue as a primary outcome. This would allow for a more targeted evaluation of how both treatments affect fatigue.

We identified disadvantages of AM in comparison to CBT-I, which are important factors when considering treatment options in a shared decision process with the patient. After 12 weeks of AM treatment participants reported more side effects than participants treated with CBT-I, most of which are common anticholinergic side effects of AM [79]. One of them was vivid dreaming, which might be the result of the REM-sleep suppressing effect of AM [80]. Despite the reported side effects, treatment adherence to AM was mostly high. However, of the participants (approximately one-sixth) who not fully completed AM treatment, most discontinued their treatment prematurely because of side effects. During both treatments, we found no elevated levels of daytime sleepiness. For CBT-I, this finding was remarkable, as CBT-I initially results in sleep deprivation, which could lead to elevated levels of daytime sleepiness [81]. In particular, we assumed that patients with medical comorbidity could experience bedtime restriction as strenuous or might be unable to fully engage in CBT-I due to their medical condition with associated disease burden. In our study, the CBT-I participants reported their adherence to the behavioral elements to be high. So, it seems that patients with insomnia and medical comorbidity are able to fully engage in CBT-I without experiencing unpleasant side effects of CBT-I.

Another disadvantage of AM was the apparent return of sleep problems after discontinuation. At 12 weeks treatment participants were requested to taper off AM. After discontinuation two-third of the participants reported worsening of sleep. This may suggest that positive effects of AM on sleep diminish after tapering. A small proportion of these participants reported this worsening of sleep as temporarily, suggesting a withdrawal effect. Besides reporting worsening of sleep, many participants reported withdrawal symptoms, such as fatigue, nervousness, headache, and nightmares.

Our findings require replication. A recently published study [82] compared low-dose AM (10–20 mg) with placebo for treating insomnia in a primary care setting. This study found a statistically significant but clinically negligible reduction in insomnia at 6 weeks, with no significant differences between AM and placebo from 12 weeks onwards. Additionally, there were no statistically significant or clinically relevant improvements in sleep (as measured by a sleep diary) or daytime functioning during or after treatment. In our study, we cannot exclude the possibility of a placebo effect with AM.

Strengths of this trial included the use of gold standard CBT-I as active comparison treatment in a noninferiority design. Furthermore, since our study was implemented in clinical care, the sample is representative for clinical practice and our RCT provides insights into how interventions function in real-world settings, which helps in understanding their effectiveness and feasibility in daily care.

Besides strengths, our study has limitations. First, there are no data on how many patients were informed about the study, as it was implemented in clinical care. The design of the study may have led to a preference for AM treatment, since CBT-I is the first-line treatment at the participating sleep–wake centers, and AM was only prescribed within the study context. Additionally, patients using antidepressants were excluded, and baseline depressive symptoms were mild, meaning the findings may not be generalizable to those with more severe psychiatric comorbidities. Furthermore, randomization was not stratified for site and medical comorbidity, which could have led to unbalanced randomization blocks and medical comorbidity distribution across sites. Another limitation is that participants were permitted to use BZRA medications during treatment, with around one-sixth of participants in both treatment groups using them, which may have affected the results. However, selecting only participants not using BZRAs during treatment did not change outcomes. The difference in waiting times between randomization and the start of treatment for the two groups (with CBT-I participants waiting about 2 weeks longer) could have influenced symptom severity or motivation in the CBT-I group. Moreover, fewer participants in the CBT-I group completed sleep diaries at 12 weeks. This may have been due to the CBT-I protocol, which requires completion of weekly diaries for the first 6 weeks, potentially leading to less adherence by the end of treatment. This lower completion rate could have impacted the subjective sleep outcomes. CBT-I was provided mostly in a group format, which has been shown to be as effective as individual treatment [69], although there is also research suggesting that group CBT-I is less effective [83]. Therefore, the results may not be generalizable to individual CBT-I treatments. Furthermore, no integrity checks on the CBT-I intervention were carried out. Finally, there were no cost-effectiveness data available for either treatment. Including these estimates would be a valuable addition to future research.

In conclusion, using a liberal non-inferiority margin, AM can be considered non-inferior to CBT-I in reducing insomnia severity in patients with medical comorbidities. However, more participants benefit from CBT-I. Furthermore, AM has more side effects and its effect on insomnia may diminish after tapering. We conclude that CBT-I remains first line treatment in patients with medical comorbidity given its broader benefits. If patients insist on medication instead of non-pharmacological treatment, AM could be considered. Then, patients should be carefully informed about potential side effects and the likely temporary effectiveness. Side effects and efficacy should be monitored during treatment. Further placebo-controlled studies testing the efficacy of AM in patients with medical comorbidity are needed.

## Supplementary Material

Supplementary_files_1-3_RCT_(1)_zsaf176

RCT_APPENDIX_A_24_12_2024_DEF_zsaf176

RCT_APPENDIX_B_24_12_2024_DEF_zsaf176

## Data Availability

Data supporting this study’s findings can be obtained from the corresponding author upon reasonable request.

## Notes

Clinical trial register: Timelapse study, July 24, 2019, URL: https://www.clinicaltrialsregister.eu/ctr-search/trial/2019-001520-35/NL.
