# Supplementary material for: Low-dose amitriptyline versus cognitive behavioral therapy for insomnia in patients with medical comorbidity: results of a randomized controlled multicenter non-inferiority trial
Source: Sleep. 2025 Jun 26;48(12):zsaf176. doi: 10.1093/sleep/zsaf176 (PMC12696364; doi:10.1093/sleep/zsaf176)
Supplement: Supplementary_files_1-3_RCT_(1)_zsaf176 [file supplementary_files_1-3_rct_(1)_zsaf176.docx]

**Low dose amitriptyline versus cognitive behavioral therapy for insomnia in patients with medical comorbidity: results of**

**a randomized controlled multicenter non inferiority trial.**

Nynke Rauwerda¹², Annemieke van Straten³, Anouk Zondervan⁴, Thom Timmerhuis⁵, Marcel Smits⁶, Pythia Nieuwkerk¹⁷, Annemarie Braamse¹⁷, H. Myrthe Boss⁶⁸ & Hans Knoop¹⁷*

1. Department of Medical Psychology, Amsterdam University Medical Center, University of Amsterdam, Amsterdam
2. Department of Medical Psychology, Hospital Gelderse Vallei, Ede, The Netherlands
3. Department of Clinical Psychology & Amsterdam Public Health Research Institute, VU University, Amsterdam, The Netherlands.
4. Department of Medical Psychology, Zaans Medical Center, Zaandam, The Netherlands
5. Department of Neurology, Jeroen Bosch Ziekenhuis, ‘s-Hertogenbosch, the Netherlands
6. Department of Neurology, Hospital Gelderse Vallei, Ede, The Netherlands
7. Amsterdam Public Health Institute, Amsterdam University Medical Center, location University of Amsterdam, The Netherlands.
8. Sleep-wake centre, Hospital Gelderse Vallei, Ede, The Netherlands

*Shared last authorship

Correspondence: N.L. Rauwerda, rauwerdan@zgv.nl

**Supplement 1.** Pre and post treatment means of daytime functioning outcomes.

| Outcome measure | Treatment | N | Pretreatment  Mean (SE) | N | Post treatment  Estimated marg.  mean (SE)^α^ | Mean between  group  difference (95% CI) | p-value  between  group diff. |
| --- | --- | --- | --- | --- | --- | --- | --- |
| **Fatigue** |  |  |  |  |  |  |  |
| CIS-fatigue | CBT-I | 94 | 41.9 (1.0) | 81 | 33.5 (1.1) | 1.5 (-1.3-4.7) | 0.27 |
|  | AM | 93 | 41.8 (1.0) | 86 | 35.1 (1.0) |  |  |
| **Physical functioning** | | | | | | | |
| SF-36-PF | CBT-I | 93 | 71.7 (2.4) | 80 | 80.2 (1.6) | 3.7 (-7.8-1.1) | 0.14 |
|  | AM | 93 | 71.7 (2.4) | 86 | 76.7 (1.6) |  |  |
| **Impairment of functioning** | | | | | | | |
| WSAS | CBT-I | 95 | 18.5 (1.0) | 67 | 11.8 (1.0) | 2.6 (-0.3-5.6) | 0.08 |
|  | AM | 93 | 18.5 (1.0) | 74 | 14.5 (1.0) |  |  |
| **Interference of bodily pain** | | | | | | | |
| SF-36-int. pain | CBT-I | 90 | 34.7 (1.5) | 80 | 38.5 (1.1) | 0.1 (-2.9-3.1) | 0.96 |
|  | AM | 92 | 36.7 (1.4) | 86 | 38.4 (1.1) |  |  |
| **Depressive symptoms** | | | | | | | |
| HADS-dep | CBT-I | 94 | 6.8 (0.4) | 81 | 4.7 (0.4) | 0.5 (-0.4-1.5) | 0.18 |
|  | AM | 93 | 6.5 (0.5) | 86 | 5.2(0.3) |  |  |
| **Anxiety** | | | | | | | |
| HADS-anx | CBT-I | 94 | 8.8 (0.2) | 81 | 8.4 (0.2) | 0.3 (-0.2-0.8) | 0.28 |
|  | AM | 93 | 9.0 (0.2) | 86 | 8.7 (0.2) |  |  |

^α^ Controlled for severity of symptom at baseline

* Checklist Individual Strenght-20, subscales fatigue severity (CIS-fat);

Short-Form 36-item Health Survey (SF-36) subscale physical functioning (SF-36=PF), subscale interference bodily pain

(SF-36-int.pain), Work and Social Adjustment Scale (WSAS) Hospital Anxiety and Depression Scale (HADS), subscale depressive symptoms (HADS_dep) and anxiety (HADS-anx)

**Supplement 2. Frequency of reported complaints on the Antidepressant Side-effect Checklist (ASEC) before and during CBT-I**

Note: Only reported complaints of participants who filled out the ASEC at all three measure moments.

**Supplement 3. Frequency of reported complaints on the Antidepressant Side-effect Checklist (ASEC) before and during AM**

Note: Only reported complaints of participants who filled out the ASEC at all three measure moments.
