# Supplementary material for: Low-dose amitriptyline versus cognitive behavioral therapy for insomnia in patients with medical comorbidity: results of a randomized controlled multicenter non-inferiority trial
Source: Sleep. 2025 Jun 26;48(12):zsaf176. doi: 10.1093/sleep/zsaf176 (PMC12696364; doi:10.1093/sleep/zsaf176)
Supplement: RCT_APPENDIX_A_24_12_2024_DEF_zsaf176 [file rct_appendix_a_24_12_2024_def_zsaf176.docx]

**Appendix A.**

Prevalence (in numbers) of medical conditions (organized in ICD-11 classification) in the CBT-I and AM participants

|  | **Prevalence** | |
| --- | --- | --- |
| **Disease ICD-11 classification** | **CBT-I** | **AM** |
| Neoplasm | 9 | 7 |
| Diseases of the blood or blood-forming organs | - | 1 |
| Diseases of the immune system | 6 | 5 |
| Endocrine, nutritional or metabolic diseases | 22 | 28 |
| Mental, behavioural or neurodevelopmental disorders | - | 1 |
| Sleep-wake disorders* | 20 | 16 |
| Diseases of the nervous system | 18 | 21 |
| Diseases of the visual system | 2 | 4 |
| Diseases of the ear or mastoid process | 2 | - |
| Diseases of the circulatory system | 13 | 7 |
| Diseases of the respiratory system | 13 | 14 |
| Diseases of the digestive system | 7 | 14 |
| Diseases of the skin | 3 | 3 |
| Diseases of the musculoskeletal system and connective tissue | 14 | 11 |
| Diseases of the genitourinary system | - | 2 |
| Developmental anomalies | 1 | 2 |
| Symptoms, signs or clinical findings, not elsewhere classified | 22 | 19 |
| Injury, poisoning or certain other consequences of external causes | 1 | - |
| Factors influencing health status or contact with health services | 1 | - |
| Codes for special purposes: new diseases of uncertain aetiology and emergency use | 1 | 2 |

*sufficiently treated breath related sleep wake disorder: n=31, Restless legs syndrome (RLS): n=5
