# Supplementary material for: Low-dose amitriptyline versus cognitive behavioral therapy for insomnia in patients with medical comorbidity: results of a randomized controlled multicenter non-inferiority trial
Source: Sleep. 2025 Jun 26;48(12):zsaf176. doi: 10.1093/sleep/zsaf176 (PMC12696364; doi:10.1093/sleep/zsaf176)
Supplement: RCT_APPENDIX_B_24_12_2024_DEF_zsaf176 [file rct_appendix_b_24_12_2024_def_zsaf176.docx]

**Appendix B.** Outcomes of the Discontinuation- Emergent Signs and Symptoms (DESS) checklist

|  | Single dosage AM  N=28 | Double dosage AM  N= 45 |
| --- | --- | --- |
|  | N(%) | N(%) |
| **Medication tapering schedule** |  |  |
| Abruptly | 15 (54) | 18 (44) |
| One week | 5 (18) | 8 (18) |
| Two weeks | 7 (25) | 16 (36) |
| Unknown | 1 (4) | 3 (7) |
|  |  |  |
| **Sleep after discontinuation** |  |  |
| Worse | 16 (57) | 28 (62) |
| Worse, temporarily | 4 (14) | 2 (4) |
| Similar or better* | 6 (21) | 13 (29) |
| Unknown | 2 (7) | 2 (4) |
|  |  |  |
| **Symptoms after discontinuation** |  |  |
| Fatigue | 5 (18) | 9 (20) |
| Nervousness | 4 (14) | 8 (18) |
| Headache | 4 (14) | 6 (13) |
| Frequently dreaming, nightmares | 4 (14) | 5 (11) |
| Restless legs | 3 (11) | 6 (13) |
| Excessive sweating | 4 (14) | 3 (7) |
| Irritability | 4 (14) | 3 (7) |
| Irritated eyes | 3 (11) | 3 (7) |
| Forgetfulness | 1 (4) | 5 (11) |
| Abdominal bloating | 3 (11) | 2 (4) |
| Muscle pain | 2 (7) | 2 (4) |
| Trembling | 2 (7) | 1 (2) |
| Difficulty concentrating / confusion | 0 (0) | 4 (9) |
| Mood swings | 0 (0) | 3 (7) |
| Agitation | 0(0) | 1 (2) |
| Diarrhea | 1(4) | 2 (4) |
| Nausea | 2(7) | 1 (2) |
| Worsening mood | 1(4) | 0 (0) |
| Episodes of crying | 0 (0) | 2 (4) |
| Muscle cramps, spasms, nerve twitches | 0 (0) | 2 (4) |
| Coordination problems | 0 (0) | 2 (4) |
| Runny nose | 0 (0) | 2 (4) |
| Stomach cramps | 0 (0) | 2 (4) |
| Blurry vision | 2(7) | 0 (0) |
| Anger outbursts | 0 (0) | 1 (2) |
| Panic attacks | 1 (4) | 0 (0) |
| Increased muscle tension | 1 (4) | 0 (0) |
| Dizziness | 1 (4) | 0 (0) |
| Shortness of breath | 0 (0) | 1 (2) |
| Cold | 0 (0) | 1 (2) |
| Restlessness | 0 (0) | 1 (2) |
| Visual sensations | 1 (4) | 0 (0) |
| Hypersensitive for noise | 0 (0) | 1 (2) |
| Tinnitus | 0 (0) | 1 (2) |
| Strange taste or smell | 0 (0) | 1 (2) |

*In the comments 4 participants reported that AM did not improve sleep and therefore after discontinuation no change in sleep was observed, and 1 reported still using BZRA.
